# Expression status and prognostic significance of mitochondrial dynamics OPA3 in human ovarian cancer

**DOI:** 10.18632/aging.204050

**Published:** 2022-05-04

**Authors:** Hsiao-Wen Tsai, Chia-Jung Li, Li-Te Lin, An-Jen Chiang, San-Nung Chen, Zhi-Hong Wen, Kuan-Hao Tsui

**Affiliations:** 1 Department of Obstetrics and Gynaecology, Kaohsiung Veterans General Hospital, Kaohsiung 813, Taiwan; 2 Institute of Biopharmaceutical Sciences, National Sun Yat-Sen University, Kaohsiung 804, Taiwan; 3 Department of Obstetrics and Gynaecology, National Yang-Ming University School of Medicine, Taipei 112, Taiwan; 4 Institute of Biomedical Sciences, National Sun Yat-Sen University, Kaohsiung 804, Taiwan; 5 Department of Marine Biotechnology and Resources, National Sun Yat-Sen University, Kaohsiung 804, Taiwan; 6 Department of Obstetrics and Gynecology, Taipei Veterans General Hospital, Taipei 112, Taiwan; 7 Department of Pharmacy and Master Program, College of Pharmacy and Health Care, Tajen University, Pingtung County 907, Taiwan; 8 Department of Medicine, Tri-Service General Hospital, National Defense Medical Center, Taipei 114, Taiwan; 9 College of Health and Nursing, Meiho University, Pingtung 912, Taiwan

**Keywords:** multi-omics, ovarian cancer, OPA3, immune infiltration

## Abstract

Early diagnosis of ovarian cancer and the discovery of prognostic markers can significantly improve survival and reduce mortality. OPA3 protein exists in a structure called mitochondria, which is the energy production center of cells, but its molecular and biological functions in ovarian cancer are still unclear. Here, the expression of OPA3 mRNA in ovarian cancer was estimated using TCGA, Oncomine, TIMER databases. We found that functional OPA3 activation caused by mutations and profound deletions predicted poor prognosis in OV patients. OPA3 was highly expressed in both OV tissues and cells compared to normal ovarian tissues/cells. High OPA3 expression is associated with poorer overall survival (OS). The association between OPA3 and immune infiltration of ovarian cancer was assessed by TIMER and CIBERSORT algorithms. OPA3 showed a strong correlation with various immune marker sets. Most importantly, pharmacogenetic analysis of OV cell lines revealed that OPA3 inactivation was associated with increased sensitivity to PFI-1, and WZ4003. Therefore, we investigated the clinical application of OPA3 to provide a basis for sensitive diagnosis, prognosis and targeted treatment of ovarian cancer.

## INTRODUCTION

With the continuous advancement of medical technology, ovarian cancer is still a troublesome cancer and frightening disease. The global incidence rate of ovarian cancer is 42 cases per 100,000 people, making it the second leading cause of death among gynecological cancers. [1, 2]. The high mortality rate makes ovarian cancer a worldwide public health issue. [3, 4]. Currently, due to the lack of sensitive tumor markers, early diagnosis of ovarian cancer is difficult work. Most ovarian cancer patients are diagnosed at an advanced stage of cancer because they came for help only when they feel abdominal pain or ascites with clinical relevance [5–7]. Therefore, the discovery of new potential targets for early diagnosis of ovarian cancer is necessary and worthy.

Metastasis is the cause of most cancer progression, leading to clinical treatment failure and patient death. Cancer cells enter the blood or lymphatic vessels through intravascular vessels. After extravasation from these vessels, they form clonal lesions in distant organs [8, 9]. Cell migration is a critical step in cancer metastasis, and several studies have demonstrated the role of mitochondria in cancer cell metastasis. For example, mitochondrial dynamics regulate the progression and distant metastasis of breast cancer cells. However, dynamic imbalances are more frequently observed in breast cancer cells with high metastasis [10]. More recently, additional evidence has emerged suggesting a regulatory role for mitochondrial dynamics in cancer metastasis [11–13]. Dysregulation of mitochondrial dynamics can lead to a malignant phenotype of cancer. Various lines of evidence link mitochondrial dynamics to cancer development and progression. Mitochondrial fusion and fission affect mitochondrial transport in lymphocytes and cancer cells. Dysregulation of mitochondrial fusion/ fission inhibits lymphocyte polarization and migration [14]. The above studies suggest that mitochondrial dynamics affect the progression of cancer.

The pathophysiology of ovarian cancer is not yet fully understood. The progression of malignant tumors is complex and involves interactions between oncogenes and the microenvironment [3, 7]. Recent studies have shown the association of oncogenes and ovarian cancers [15]. The optic atrophy 3 (OPA3) gene was first identified in patients with optic neuropathy, and OPA3 is located in the mitochondria and has a biological function in maintaining the shape and structure of the mitochondria. Previous study had found overexpression of OPA3 may cause mitochondria fragmentation and knockdown of OPA3 cause elongation of mitochondria in ARPE-19 cells [16]. Since the interaction between oncogenes and ovarian cancer is a key factor in tumor development, we tried to identify the role of OPA3 in ovarian cancer.

Therefore, our current study is dedicated to explore the impact of OPA3 in the diagnosis and prognosis of ovarian cancer patients. We further explored the role of OPA3 in ovarian cancer by systematically analyzing OPA3 expression and analyzing the potential mechanisms in the malignant transformation of ovarian cancer in multiple publicly available databases.

## MATERIALS AND METHODS

### Oncomine

The Oncomine platform is a publicly accessible online tumor-associated gene microarray database for collecting relevant gene expression profiles and related clinical information [17]. The Oncomine database includes approximately 200 biopsies analyzing OPA3 gene transcript levels in different tumors and normal tissues.

### cBioPortal

cBioPortal is used to perform interactive analysis of biomolecules in tumor tissues in the TCGA database. We used cBioPortal to mine gene set data and ovarian cancer gene variants. A hypothetical copy number of 370 cases identified using GISTIC 2.0 was deployed. In module comparison/survival, we analyzed OPA3 mutations, copy number variants (CNV) and gene coexistence in ovarian cancer.

### GEPIA2

OPA3 expression data in various human tumor tissues were obtained from the Gene Expression Profiling Interactive Analysis (GEPIA) public database [18, 19]. GEPIA2 was used to compare OPA3 expression levels in OV, and we used default parameters to analyze OPA3 expression at different OV stages.

### Cells and cell culture

In this study, multiple human ovarian cancer cell lines including: OC-117-VGH cells, OC-117-VGH cells, OCPC-2-VGH cells, OC-3-VGH cells, TOV-21G cells and NIH-OVCAR-3 cells (BCRC#6060, #60602, #60603, #60599, #60407, #605511, Hsinchu, Taiwan) were cultured in a humidified atmosphere supplemented with 1.5 g/L sodium bicarbonate and 10% FBS (ThermoFisher Scientific, MA, USA) at 37° C in 95% air and 5% CO_2_.

### RNA extraction and real-time PCR

RNA isolation of all samples was performed using EasyPrep Total RNA Kit (BIOTOOLS Co., Ltd., Taipei, Taiwan.), as indicated above. Next, 1 μg of total RNA was reverse transcribed using a ToolScript MMLV RT kit. (BIOTOOLS Co., Ltd.) in an Applied Biosystems™ (ABI 7500) under the following reaction conditions: 65° C for 5 min, 42° C for 60 min, and 70° C for 10 min. The resulting cDNAs were subjected to quantitative real-time PCR (qRT-PCR) analysis using a TOOLS 2X SYBR qPCR Mix (BIOTOOLS Co., Ltd.) in a StepOnePlus Real-Time PCR system. The conditions used included an initial step at 95° C for 10 min, followed by 40 cycles at 95° C for 15 s and a final step at 60° C for 1 min. Ct values were calculated using U6 (RNU6-1) as reference. Untreated samples were used as controls to determine the relative fold-changes in mRNA expression.

### Human BRCA tissue microarray (TMA) and immunohistochemistry (IHC) analysis

The IHC staining and scoring in this study was the same as our previously published method. We analyzed OPA3 expression on tissue array slides (CJ2, SuperBioChips Laboratories, Korea) purchased from human ovarian cancer and measured and scored immunohistochemistry (IHC) as described in a previous publication [20]. To detect protein levels, *in situ* hybridization was performed with OPA3 antibody (A4995, ABclonal, MA, USA).

### Human Protein Atlas

We validated OPA3 levels in normal and tumor tissues using the Human Protein Atlas public database. This database is designed to map the biology of all human proteins in cells, tissues and organs by integrating various histological techniques including antibody-based imaging, mass spectrometry-based proteomics, transcriptomics and systems.

### Tumor immune estimation resource (TIMER)

TIMER is a web-based resource for the systematic evaluation of the clinical impact of different immune cells on different cancer types. We used this database to compare OPA3 expression with changes in the ovarian tumor microenvironment [21]. T We selected OPA3 in the TIMER database as an input for the detection of ovarian cancer under the Immune Association Module and analyzed the correlation of OPA3 with other immune cells.

### Statistical analyses

All data are shown as the mean ± S.E.M. In the case of single mean comparisons, data were analyzed by Student’s t-tests. For multiple comparisons, statistical difference was calculated by one-way ANOVA. ***Post hoc*** analysis was performed with Tukey’s test when one-way ANOVA showed significant differences. Statistical analyses were carried out using Prizm 8.0 (GraphPad Software). *p value < 0.05; **p value < 0.01; ***p value < 0.001.

### Data availability statement

The dataset supporting the conclusions of this article is included within the article.

## RESULTS

### Characterization, mutation and copy number changes of OPA3 gene in OV

**Figure 1:**
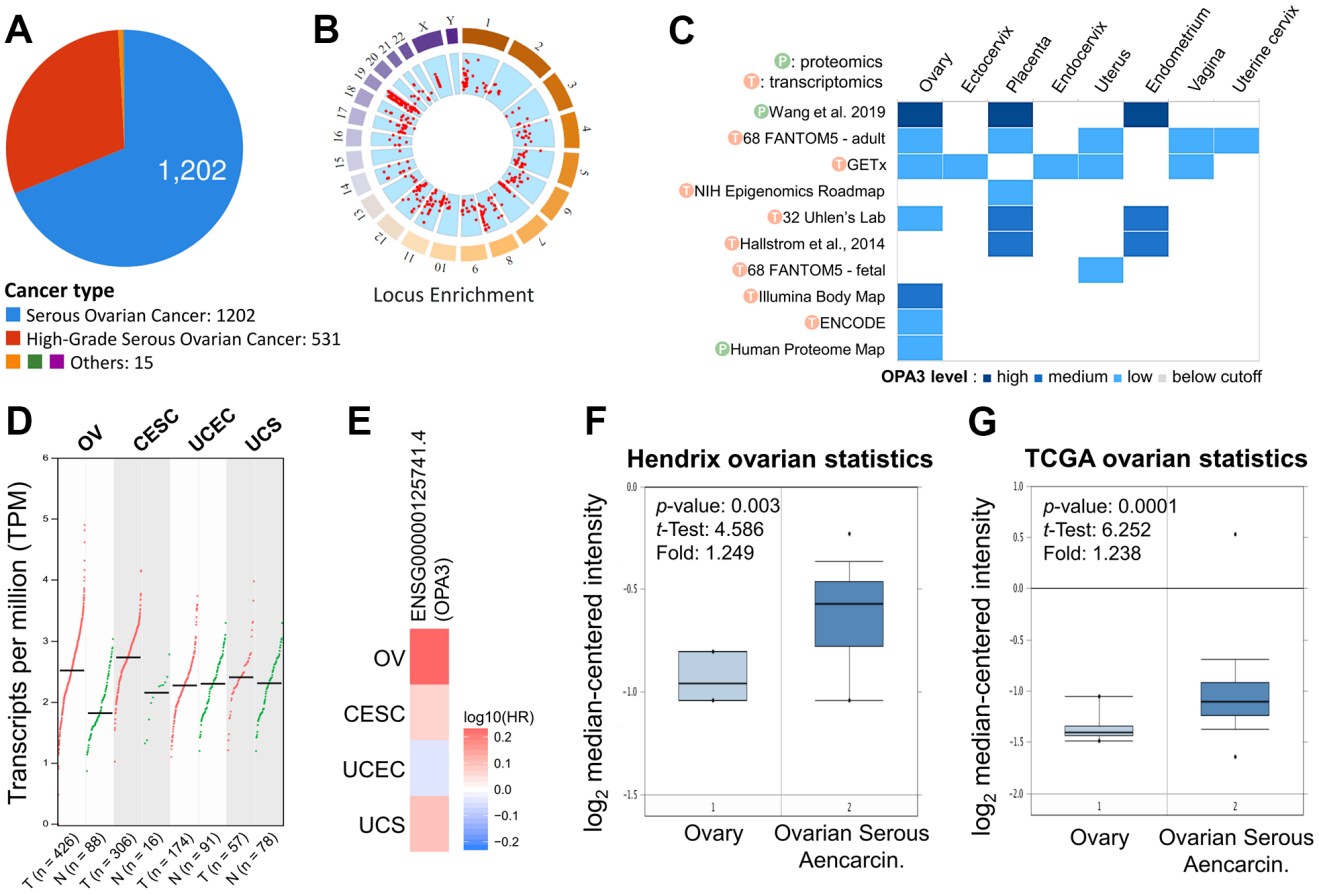
**Expression analysis with OPA3 in the reproductive system.** (**A**) Percentage of each ovarian cancer type in the TCGA dataset from cBioPortal. (**B**) Percentage of mutation counts and genomic changes due to copy number changes for different ovarian cancer types in the TCGA dataset. (**C**) RNA and protein levels of OPA3 in the reproductive system in the GETx database. (**D**, **E**) Gene transcriptional expression and survival of OPA3 in the female reproductive system. (**F**, **G**) Box plots of OPA3 mRNA levels in OV and normal tissues from Hendrix and TCGA ovarian Statistics.

To identify changes in potential oncogenes in OV in the TCGA dataset. Among ovarian cancers, the majority of tumors were serous ovarian cancer (n = 1202) and high-grade serous ovarian cancer (n = 531) (Figure 1A). The red dots in the “Locus Enrichment” diagram represent a gene and its associated location on the chromosome. The correlation between RNA level and CNV for each gene (Figure 1B). The transcriptional and translational expression levels of OPA3 in the reproductive system showed a high expression of OPA3 in the ovary (Figure 1C). We further analyzed the results from the cBioPortal database and showed that OPA3 expression in ovarian and cervical cancers was most significant in female reproductive cancers (Figure 1D). The same results were reflected in the survival rate in the ENSG00000125741.4 database, where the HR was similarly increased in ovarian cancers with high OPA3 expression (Figure 1E). In addition, OPA3 mRNA level was significantly higher in ovarian cancer than in normal tissue in both Hendrix and TCGA databases (Figure 1F, 1G).

### The genetic alteration landscape of OPA3 in OV

**Figure 2:**
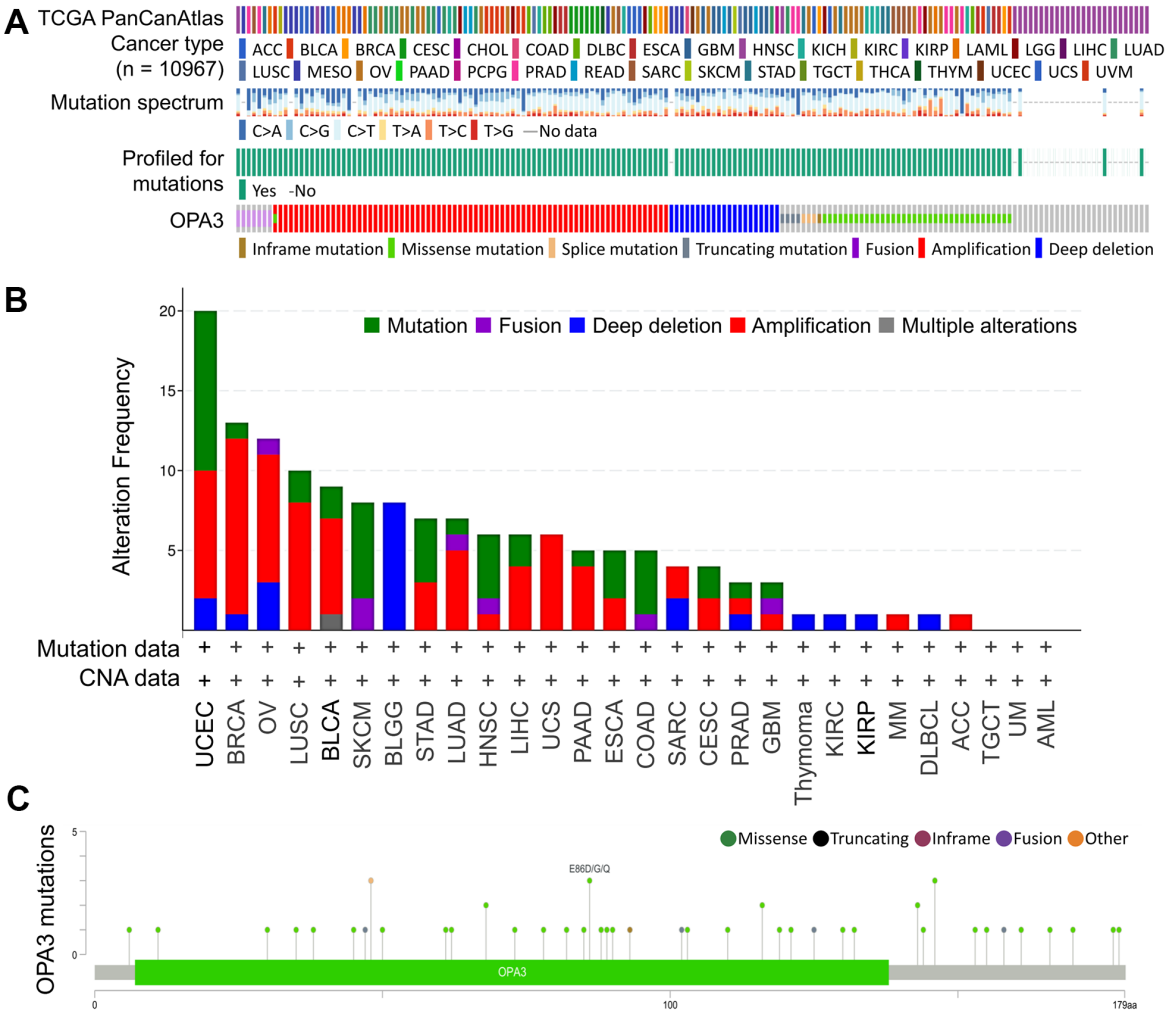
**Analysis of OPA3-related transcription factor variants in ovarian cancer.** (**A**) Frequency and type of OPA3 gene mutations in pancancer. (**B**) Distribution of copy number mutations of pan-oncogenes from TCGA. (**C**) OPA3 protein structural domain and location of specific mutations. The length of the line linking the mutation annotation to the protein indicates the number of samples with mutations.

We then used the cBioPortal database to evaluate the mutation and frequency of OPA3 in OV tissues based on data from the Pan-Cancer Atlas database of OV patients obtained from TCGA. We found that 3% of OPA3 genes were mutated in various cancers (Figure 2A). We examined the genetic alterations of OPA3 in various tumor types in the TCGA dataset. We found that the top three most frequent OPA3 gene alterations were in cervical cancer tumors, BRCA, and ovarian cancer (Figure 2B), which also indicates that OPA3 is more frequently mutated in female cancers. We further explored the specific changes in each gene, with the highest number of mutated sites in the core structure of OPA3 (Figure 2C). The somatic cell copy alteration (sCNA) module allows users to compare the distribution of different cancer types by the sCNA status of genes from the TCGA database. For this purpose, we examined the ratio of Arm-level deletion, Diploid/Normal, and Arm-level gain of OPA3 genes.

**Figure 3:**
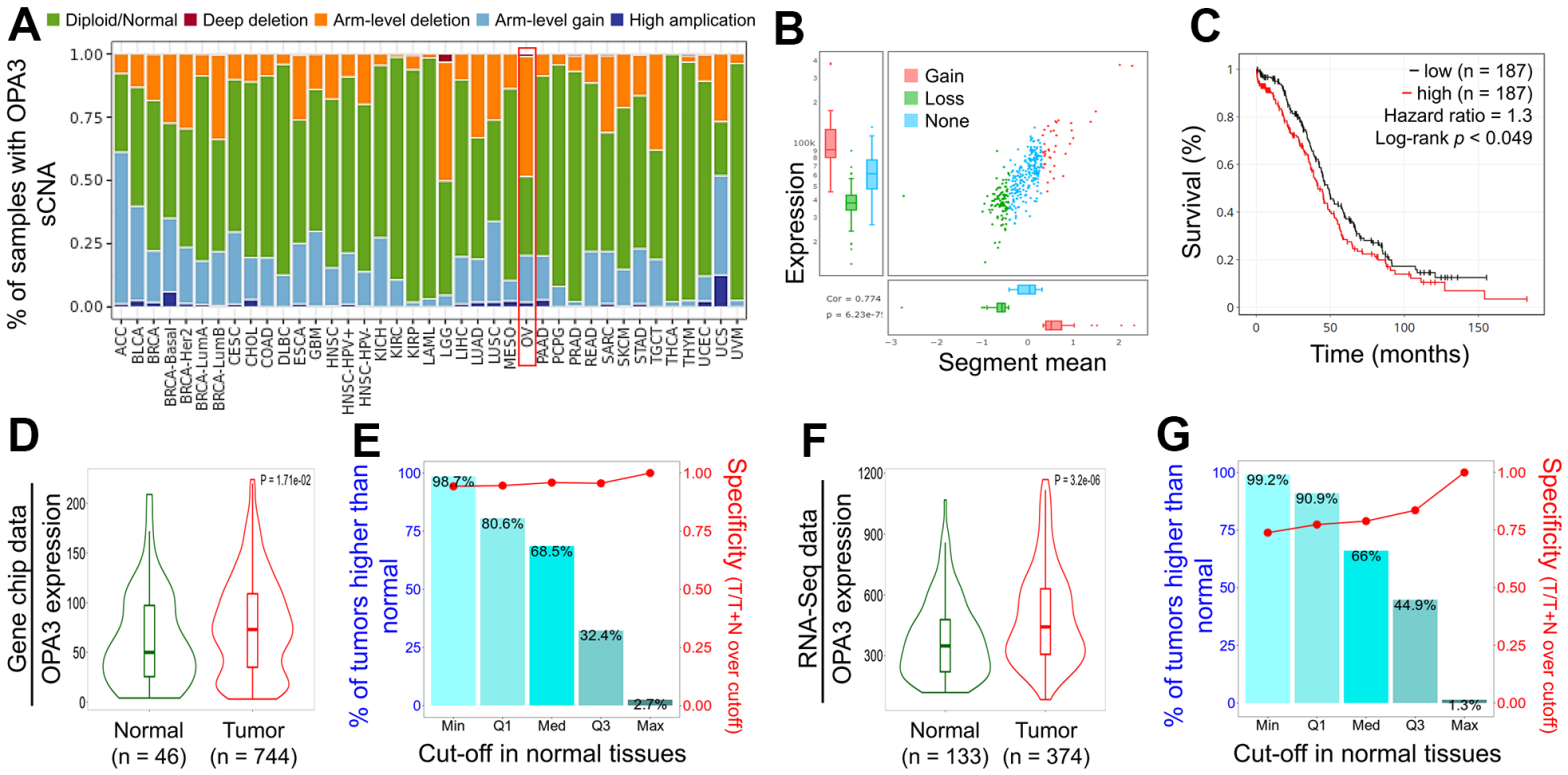
**Relative expression and survival of OPA3 in OV tissues based on multiple databases.** (**A**) Illustration of the definition of somatic cell copy alteration (SCNA) at the OPA3 gene level.(**B**) Distribution and correlation of CNV in ovarian cancer are marked in red (gain) and green (loss) to visualize the distribution of log2 ratios. (**C**) Overall survival estimates of OPA3 mRNA levels from the Kaplan-Meier Mapper database. Violin plots (**D**, **F**) and box plots (**E**, **G**) of OPA3 gene expression from RNA sequencing data and gene microarray data.

We observed a high “Arm-level deletion” of OPA3 master regulators in ovarian cancer (Figure 3A). Next, we observed significant changes in OPA3 gain and loss in the CNV ratio distribution and box plot (cor. = 0.774) (Figure 3B). While OV patients were divided into low and high OPA3 groups using median expression thresholds, the expression difference between the two groups was significant (p < 0.049) in Figure 3C. Kaplan-Meier survival analysis showed a shorter survival rate in the high OPA3 group than in the low OPA3 group (Figure 3C). We used TNM plots to analyze OPA3 expression from gene microarray data and RNA-seq data (Figure 3D, 3F) (*p* = 1.71e-02, *p* = 3.2e-06). We also analyzed the specificity of OPA3 in OV from both data (gene chip and RNA-seq.). OPA3 expression in tumor biopsies was higher than the percentage of normal biopsies at each of the major cut-off values in Figure 3E, 3G.

### Protein expression levels of OPA3 in OV patients

**Figure 4:**
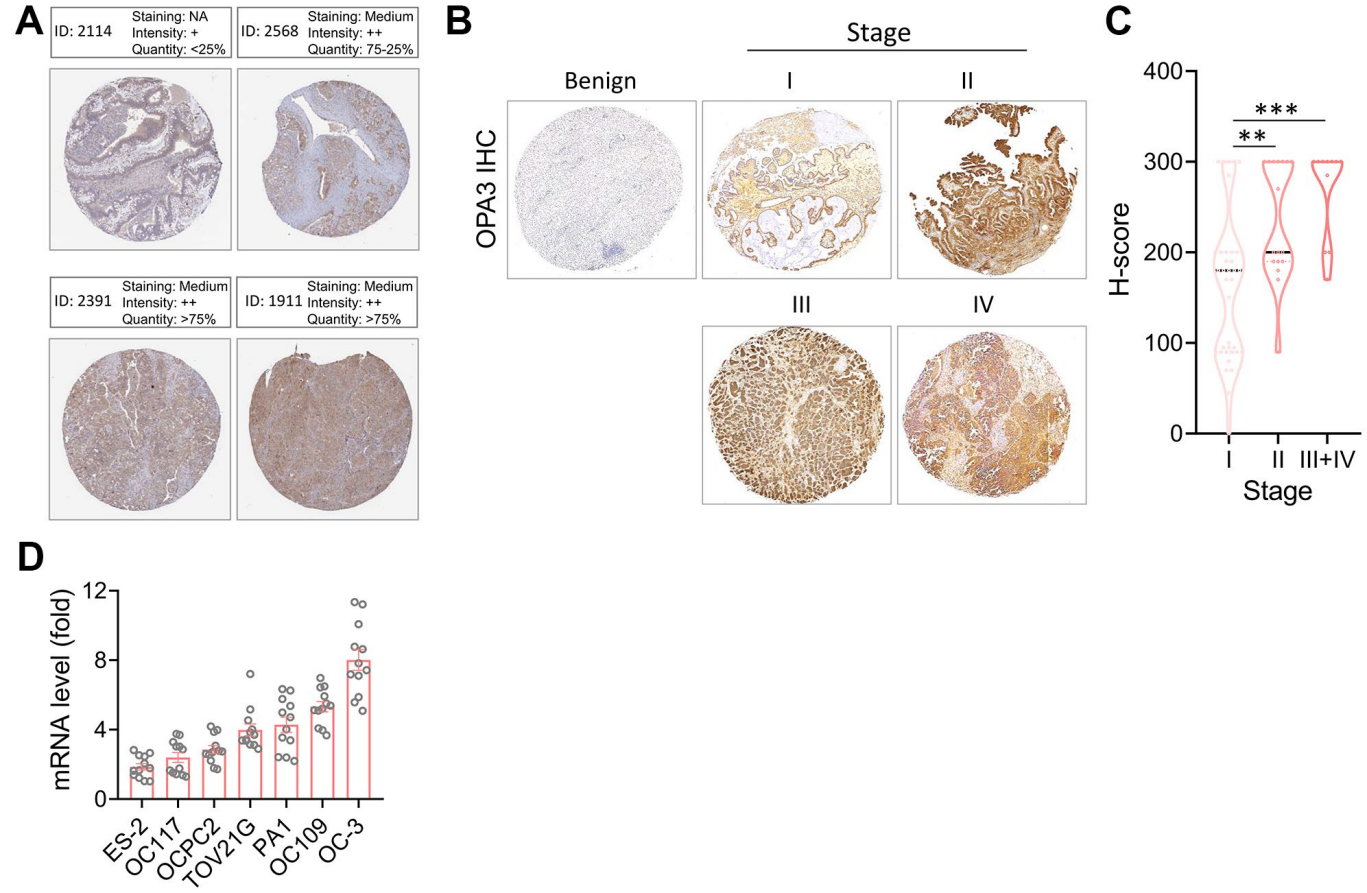
**Protein levels of OPA3 in ovarian carcinoma.** (**A**) Representative images of OPA3 IHC staining in ovarian cancer from the human Protein Atlas dataset. (**B**) Representative images of OPA3 expression at different stages of OV. (**C**) Violin plots of OPA3 expression levels OV in different stages. (**D**) RT-PCR was used to detect the expression levels of different ovarian cancer cells. ** p < 0.01., *** p < 0.001 Scale bar = 500 μm.

First, we identified OPA3 in OV patients through the Human Protein Atlas (HPA) database showing moderate staining levels (Figure 4A). Next, to further evaluate the accuracy of the HPA database, we used immunohistochemistry to assess the protein levels of OPA3 detected in TMA using human ovarian cancer tissue microarray (TMA) at different stages. Figure 4B shows the results of OPA3 protein expression in IHC-stained ovarian cancer tissues. The H-score of OPA3 showed a positive correlation increase with stage, and OPA3 protein expression was significantly higher in stage I-IV patients than in benign patients at different stages. OPA3 expression was higher in stage III+IV than in stages I and II (Figure 4C). The analysis of clinical biopsies confirmed the same trend as the above-mentioned multi-histological database, with higher OPA3 expression in advanced stages of ovarian cancer. We also tested the endogenous levels of OPA3 in multiple ovarian cancer cell lines and the data indicated that OPA3 expression in various ovarian cancer cell lines was positively correlated with higher malignancy (Figure 4D).

### Analysis of differentially expressed genes correlated with OPA3 in OV

**Figure 5:**
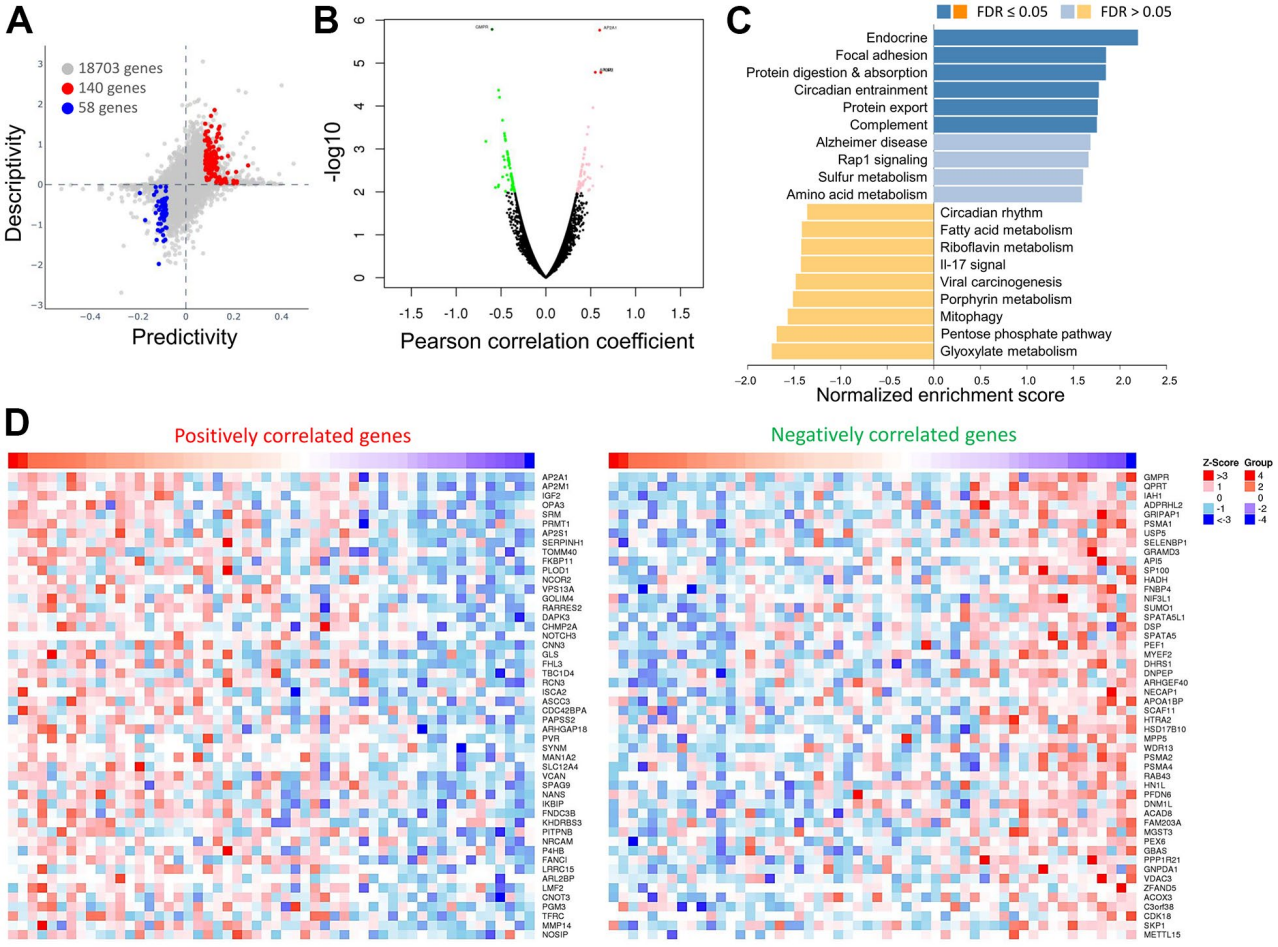
**Comparisons and enrichment analysis of gene expression profiles in OPA3 in OV.** (**A**) The predictability and descriptiveness between mRNA expression functions are plotted with ovarian cancer cell lines. (**B**) Analysis of differential gene expression associated with OPA3 in OV. (**C**) Functional enrichment analysis of OPA3 in OV. (**D**) Heatmap showing the top 50 genes are each significantly positively and negatively correlated with OPA3.

Next, we attempted to show the association between the efficacy of OPA3 in ovarian cancer and the expression of the target genes. We used statistical tests to calculate the two-way predictive and descriptive scores for each of the more than 16,000-17,000 genes. The results in Figure 5A indicate that 140 genes (red dots) had positive predictive and descriptive scores, while 58 genes (blue dots) had negative scores. The volcano map shows the genes positively and negatively associated with OPA3 (Figure 5B). The red dots are the clusters of genes positively associated with OPA3, while the green dots are the clusters of genes negatively associated with OPA3 (p < 0.01 and FDR < 0.01). We enriched the genes with more significant differences and classified them according to their biological functions in Figure 5B. The top 20 significant gene clusters positively and negatively associated with OPA3 by functional enrichment are shown (Figure 5C). The heat map shows the top 50 positively and 50 negatively associated genes with the broad effect of OPA3 on the transcriptome (Figure 5D).

### Correlation of OPA3 expression with tumor microenvironment

**Figure 6:**
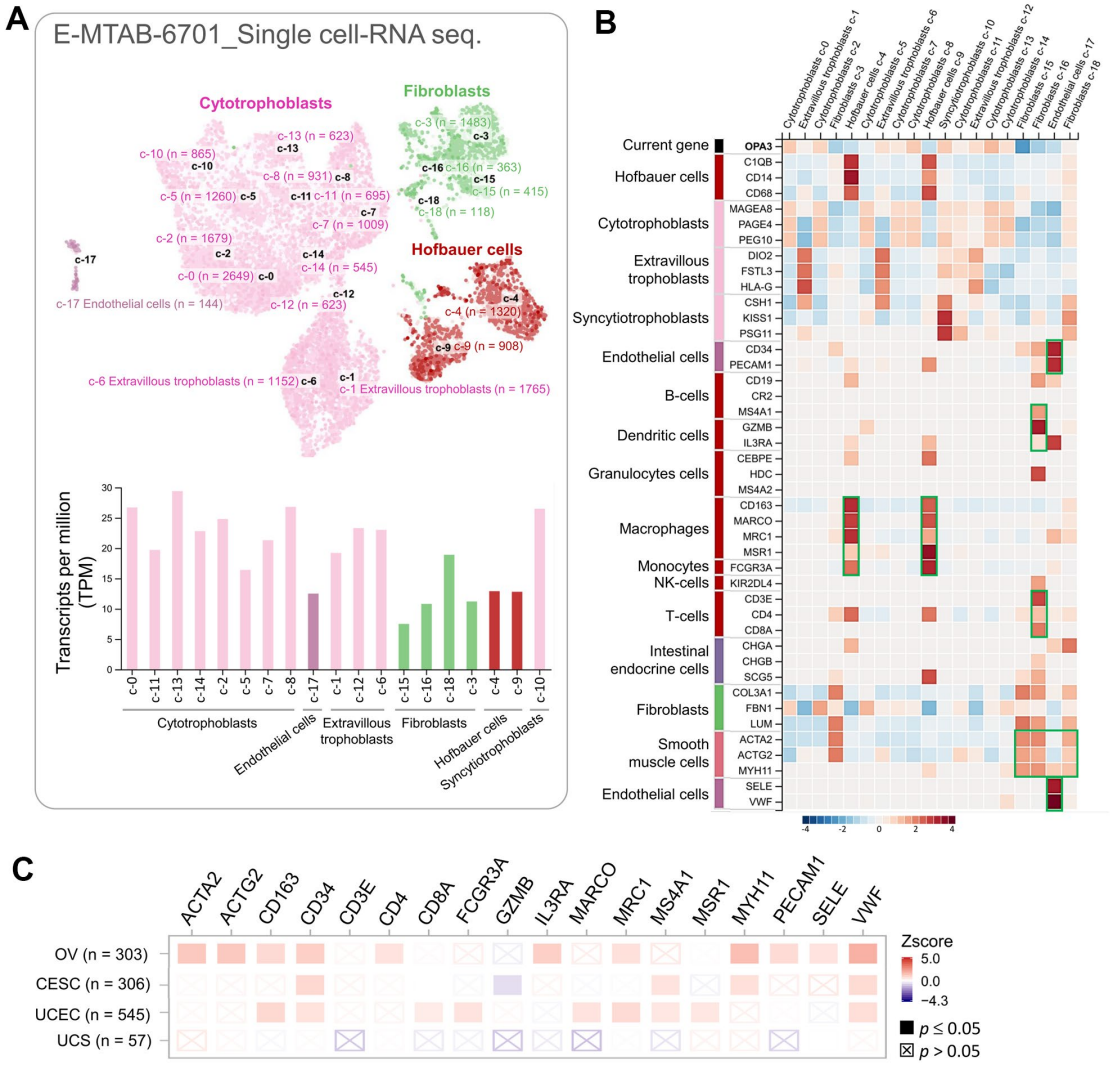
**The correlation between OPA3 and immunization.** (**A**) Single cell-RNA sequencing in identified single cell type clusters in placental tissue as shown by UMAP plots and bar graphs. (**B**) Heatmap showing the expression of OPA3 gene and well-known cell type markers in different single cell type clusters of the tissue. (**C**) Relationship between immune cell infiltration and OPA3 expression.

We used single-cell RNA sequencing datasets to analyze transcriptomic data and to clarify heterogeneous cell populations and reconstruct the microenvironment of cell development. The HPA database (10× Genomics) was used to explore the potential association of OPA3 with immune cells in the ovarian microenvironment. We obtained 18,547 single cells in this database as UMAP plots and bar graphs (Figure 6A). The specificity and distribution of OPA3 in different cell populations in the ovary were analyzed to determine the variation in gene number in single-cell types. The heatmap shown in the left panel indicates the expression of different biomarkers in multiple cell clusters in the ovarian tissue. We identified specific immune cells (green boxes) with high expression from the single-cell sequencing data (Figure 6B). Therefore, we further analyzed OPA3 and immune highly correlated genes in female reproductive cancers through different databases with highly correlated immune markers in Figure 6B. The results showed that OPA3 was highly associated with these immune signatures in ovarian cancer among female reproductive cancers (Figure 6C).

### Relationship between OPA3 and different immune marker sets

**Figure 7:**
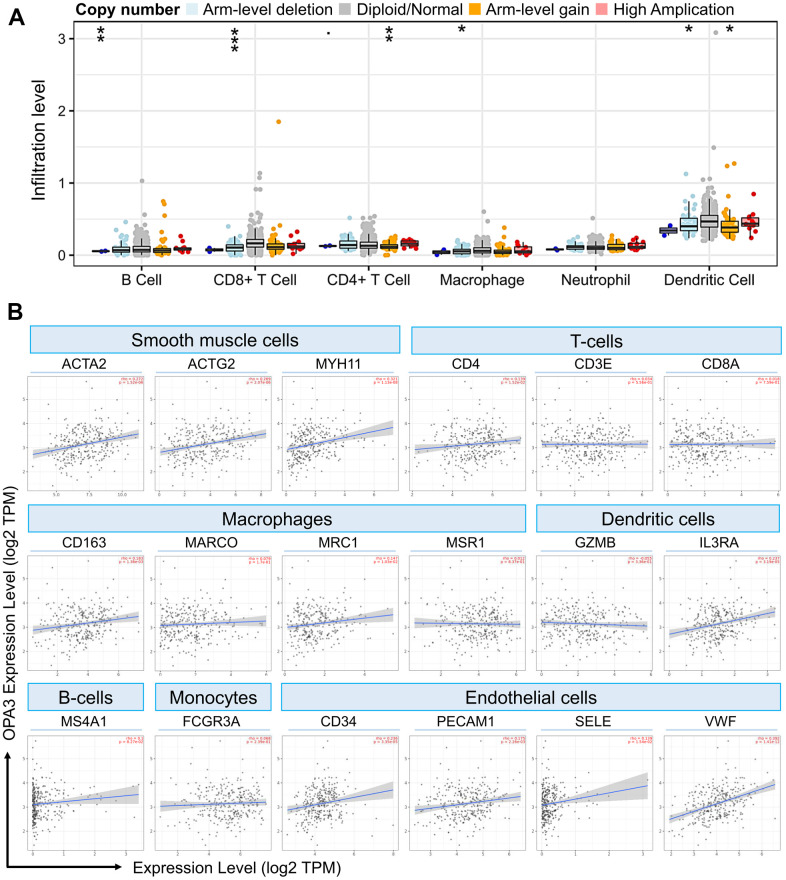
**OPA3 is closely related to immunity in OV.** (**A**) OPA3 copy number variable (CNV) affects infiltration levels of CD8+ T-cells, macrophages, neutrophils, and dendritic cells in OV. (**B**) Relationship between immune cell infiltration and OPA3 expression.

Our results from the TIMER analysis of the immune database showed that the absence or expansion of different forms of OPA3 copies was associated with the possibility of differential suppression of immune cell infiltration by many immune cells (Figure 7A). In ovarian cancer, OPA3 expression was associated with different types of immune cells. As shown in Figure 7A, Arm-level deletion of OPA3 was associated with B cells and CD8+ T cells, macrophage and dendritic cells; Arm-level gain of OPA3 was associated with CD4+ T cells and dendritic cells. We observed high expression levels of several cells in ovarian cancer in Figure 6 single-cell sequencing. Thus, we found that endothelial cells and dendritic cells are the two immune cell types most closely correlated with OPA3 (Figure 7B). We further dissected the correlation between OPA3 and the immune biomarkers of smooth muscle cells, tumor-associated macrophages (TAM), M1 macrophages and M2 macrophages. OPA3 was positively correlated with smooth muscle cell infiltration in OV tissues but not in normal ovarian tissue.

### Pharmacogenetic screening for potential drugs to inhibit OPA3 in OV

**Figure 8:**
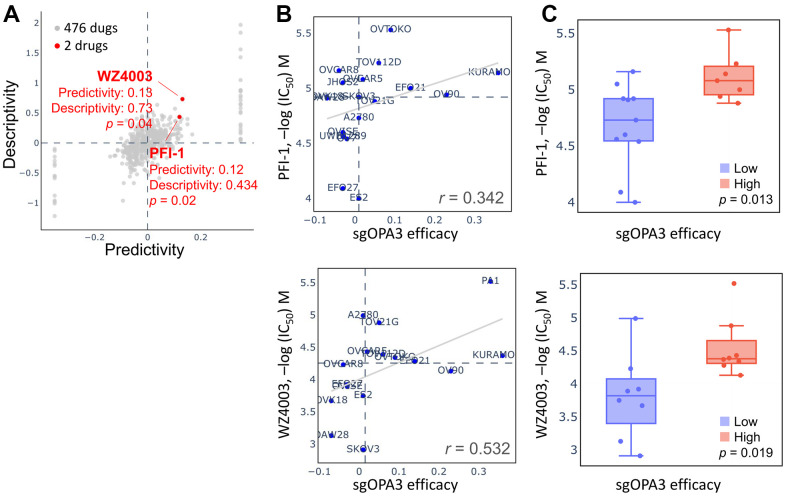
**Drug sensitivity and cytotoxicity analysis in ovarian cancer cells.** (**A**) Use of the database to query OPA3 gene signatures and screen for potential drugs. (**B**) Drug sensitivity of sgOPA3 gene to PFI-1 and WZ4003 in OV cell lines. (**C**) OPA3 efficacy of PFI-1 and WZ4003 in inhibiting OV cancer cells.

We attempted to retrieve the OPA3 gene library from the pharmacogenetic database for screening potential drugs for the therapy of ovarian cancer. As shown in Figure 8A, two of the 476 drugs were significant, including PFI-1 and WZ4003, which inhibit OPA3 overexpression. When queried in the Q-omics database for the relationship between PFI-1 and WZ4003 in knocking down OPA3 co-expression gene features, we found that the relationship between PFI-1 and WZ4003 for CRISPR OPA3 knockdown We found a high sensitivity and positive correlation between PFI-1 and WZ4003 for CRISPR OPA3 knockdown (PFI-1: r = 0.342; WZ4003: r = 0.532) (Figure 8B, 8C). Therefore, PFI-1 and WZ4003 have anti-cancer potential to inhibit the growth of ovarian cancer cells with high OPA3 expression.

## DISCUSSION

Cancer is a disease characterized by abnormal cell proliferation and metabolic dysregulation [22]. However, previous studies have pointed out that mitochondrial dynamics have been shown to be related to these mechanisms, and evidence suggests that mitochondrial fusion and fission processes are closely associated with cancer progression in different types of cancer [23]. Mitochondrial dynamics balance in cancer cells plays a key role in cellular biogenesis and maintenance of mitochondrial morphology and antioxidation. The key to change in mitochondrial morphology is the fusion and fission in dynamic equilibrium to maintain the constant level of intracellular nutrients [24]. In addition, cancer metabolism refers to the altered cellular metabolic pathways evident in cancer cells compared to normal cells and to the reprogramming and utilization of nutrients in the tumor microenvironment to increase the nutrient enrichment of cancer cells during metastasis and progression [25].

The growth of cancer cells requires suitable nutritional supplementation, therefore, cancer cells with high fatty acid synthesis rate and glycolytic activity in proliferating rapidly [26]. Metastatic cancer cells respond by increasing metabolic turnover rate to maintain the energy required for distant metastasis [27, 28]. In general, fragmented mitochondria have lower oxidative metabolic activity compared to tubular mitochondria. In contrast, the limited mitochondrial oxidation preserves glycolytic metabolites that can be used as a resource for cancer cell proliferation. Mitochondrial fission has also been associated with highly activated glycolysis in a variety of cancers [29].

Previous studies have noted that inhibition of DRP1 or in combination with chemotherapy reduces cancer cell proliferation and/or programmed cell death in several cancer types observed *in vitro* and *in vivo*, including pancreatic, colon, lung, hepatocellular carcinoma, and melanoma [30–33]. Furthermore, focusing on related proteins involved in mitochondrial dynamics are considered potential biomarkers of malignancy or therapeutic targets in different tumor types. Thus, reduced fragmentation of mitochondria is correlated with genomic instability in breast cancer cell lines that normally have high levels of globular mitochondria [34–36]. The underlying mechanisms of cellular dysfunction and cell death induced by mitochondrial dynamic imbalance appear to be factors that severely affect genomic replication, oxidative stress, and mitotic abnormalities in cancer cells. However, once mitochondrial dynamics are imbalanced, mitochondrial dysfunction can also result, including mtDNA mutations and excessive accumulation of ROS. This genomic instability and abnormal mitochondrial function ultimately leads to the initiation of the mitochondrial apoptotic pathway. This also suggests that reducing excessive mitochondrial fission in cancer cells may enhance apoptosis, increase therapeutic sensitivity, and increase therapeutic efficacy by contributing to reduced drug tolerance in cancer cells [37].

There are few studies related to OPA3 and various cancers, and few studies have been conducted on this topic, it is an issue worth exploring in depth. Mitochondrial dynamics are balanced to enable cells to cope with environmental changes and metabolic reprogramming, such as tumor development and remote cancer metastasis. OPA3 may promote cellular energy metabolism, and its upregulation in K-ras-driven cancers may be a mechanism to counteract the negative effects of K-ras on mitochondria to maintain energy homeostasis [38]. Thus, OPA3 may play an important role in ovarian cancer and has the potential to become a therapeutic target or biomarker. This study observed a high level of differential OPA3 gene amplification in pan-cancer. However, gene amplification is a process by which the genome of a specific protein-encoded gene selectively increases while other genes do not increase proportionally.

Finally, we constructed a multi-omics study to characterize OPA3 in ovarian cancer to provide a good predictor for prognosis. Single cell sequencing and network analysis confirmed the regulatory relationship and interaction between immunity and OPA3, which could also serve as a basis for future immunotherapy. However, although we provide clinical data to support our view, the insufficient sample size remains a limitation of this study. Moreover, future prospective and validation studies are necessary to perform to predict the accuracy of genetic features.

## CONCLUSIONS

In summary, we comprehensively analyzed the prognostic value and expression of OPA3 in human ovarian cancer. The high expression of OPA3 indicated that the prognosis of ovarian cancer was poor. Whether inhibition of the key player OPA3 could serve as a diagnostic basis or therapeutic target for prostate cancer and could more comprehensively inhibit the progression of the tumor microenvironment. Of course, further experiments are needed to test these hypotheses. There are relatively few studies on OPA3 in ovarian cancer. Our work may lay the foundation for future exploration of the biological function of OPA3 in tumors and its impact on clinical drug resistance.
